# Fabrication of biocompatible and mechanically reinforced graphene oxide-chitosan nanocomposite films

**DOI:** 10.1186/1752-153X-7-39

**Published:** 2013-02-25

**Authors:** Ping-Ping Zuo, Hua-Feng Feng, Zhi-Zhen Xu, Ling-Fan Zhang, Yu-Long Zhang, Wei Xia, Wen-Qing Zhang

**Affiliations:** 1 School of Chemistry and Molecular Engineering, East China University of Science and Technology, 130 Meilong Road, Shanghai, 200237, China

**Keywords:** Graphene oxide, Chitosan, Chemical modification, Biocompatibility, Biofilms

## Abstract

**Background:**

Graphene oxide (GO)can be dispersed through functionalization, or chemically converted to make different graphene-based nanocomposites with excellent mechanical and thermal properties. Chitosan, a partially deacetylated derivative of chitin, is extensively used for food packaging, biosensors, water treatment, and drug delivery. GO can be evenly dispersed in chitosan matrix through the formation of amide linkages between them, which is different from previous reports focusing on preparing GO/chitosan nanocomposites through physical mixing.

**Results:**

In this study, free-standing graphene oxide-chitosan (GO-chitosan) nanocomposite films have been prepared. The GO-chitosan films are biologically compatible and mechanically reinforced. Through the formation of amide linkages between GO’s carboxylic acid groups and chitosan's amine groups, GO could be evenly dispersed within the chitosan matrix. We also characterized the GO-chitosan composite films using element analysis, Fourier transform infrared spectroscopy, X-ray photo electron spectroscopy, differential scanning calorimetry, and thermo gravimetric analysis. Compared to pristine chitosan film, the tensile strength of GO-chitosan film is improved by 2.5 folds and Young’s modulus increases by nearly 4.6 folds. The glass transition temperature of GO-chitosan composite film shifts from 118°C to 158°C compared to the pristine chitosan, indicating its enhanced thermal stability. GO-chitosan composite film was also evaluated for its biocompatibility with C3H10T1/2 cells by in vitro fluorescent staining. The graphene oxide-reinforced chitosan composite films could have applications in functional biomaterials.

**Conclusion:**

The present study describes a useful and simple method to chemically attach biocompatible chitosan onto graphene oxide. We envision that the GO-chitosan film will open avenues for next-generation graphene applications in the realm of functional biomaterial.

## Background

Since its discovery, graphene has attracted considerable attention due to its fascinating properties [1-4], as evidenced by a substantial amount of primary literature. Perfect two-dimensional single-layered graphene does not exist naturally, but bulk and solution processable functionalized graphene materials, including graphene oxide (GO) have been successfully prepared [5]. The polar functional groups attached on the basal planes and at the edges of GO sheets alter their properties significantly [6]. Combined, these altered properties provide convenient access for fabrication of graphene-based materials by solution casting, and can form large-scale uniform films on various substrates. Recent studies [6-8] have shown that GO can be dispersed through functionalization, or chemically converted to make different graphene-based nanocomposites with excellent mechanical and thermal properties. Graphene-based sheets have also been tested as possible nanocarriers for delivering drugs [9] and also as functional biomaterials [10,11]. Shen et al. reported graphene oxide-based biocomposites through diimide-activated amidation and found that the covalently bonded biomaterials retained their bioactivity [12], while Dai et al. reported non-toxic PEGylatednano-graphene oxide could delivery water-insoluble cancer drugs [13,14].

**Figure 1:**
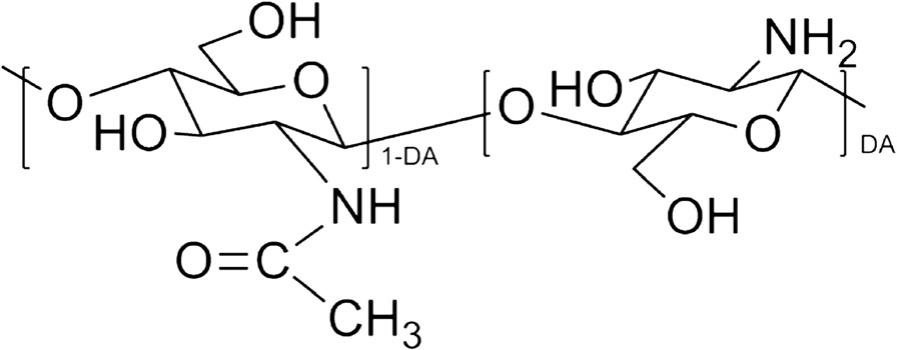
**The structure of partially deacetylated chitosan.** DA means a copolymer characterized by its average degree of deacetylation.

Chitosan, a partially deacetylated derivative of chitin, is a linear polysaccharide consisting of *β* (1,4)-linked D-glucosamine residues (deacetylated unit) with a variable number of randomly located N-acetyl-glucosamine groups (acetylated unit) (Figure 1). As one of the most abundant natural biopolymers on earth, it is extensively used for food packaging, biosensors, water treatment, and drug delivery [15]. It has been reported that chitosan-based biomaterials could promote chondrogenesis [16].

Despite its unique physical properties, the mechanical properties of pristine chitosan are not good enough to satisfy a wide range of applications.

Here we demonstrate that GO can be evenly dispersed in chitosan matrix through the formation of amide linkages between them, which is different from previous reports focusing on preparing GO/chitosan nanocomposites through physical mixing [17-21]. Since the chitosan can provide a biocompatible, transferable, and implantable condition for cell culture [22,23], we also evaluate the viability of cells on GO-chitosan composite film using C3H10T1/2 cells, for potential applications in growth of articular cartilage [24].

## Results and discussion

ElementarVario EL III was used to analyse the mole ratio of chitosan to GO in the GO-chitosan composite. The amount of N-element in chitosan is about 1:1 (N:chitosan, mol/mol). The elemental analysis shows that GO-chitosan is composed of 7.83 wt% of nitrogen, 42.54 wt% of carbon, 6.61 wt% of hydrogen and 43.02 wt% of oxygen. Based on the results, we can calculate the amount of chitosan in GO-chitosan is 90.05 wt% and the amount of GO is 9.95 wt%. The mass ratio of GO to chitosan is 1:9 in GO-chitosan compound.

To compare the same component in two different films, the amount of GO is 10.0% weight in the mixture preparation. The GO/chitosan mix solution contains 0.25 wt% GO and 2.25 wt% chitosan, and the mass ratio of GO to chitosan is 1:9, too.

**Figure 2:**
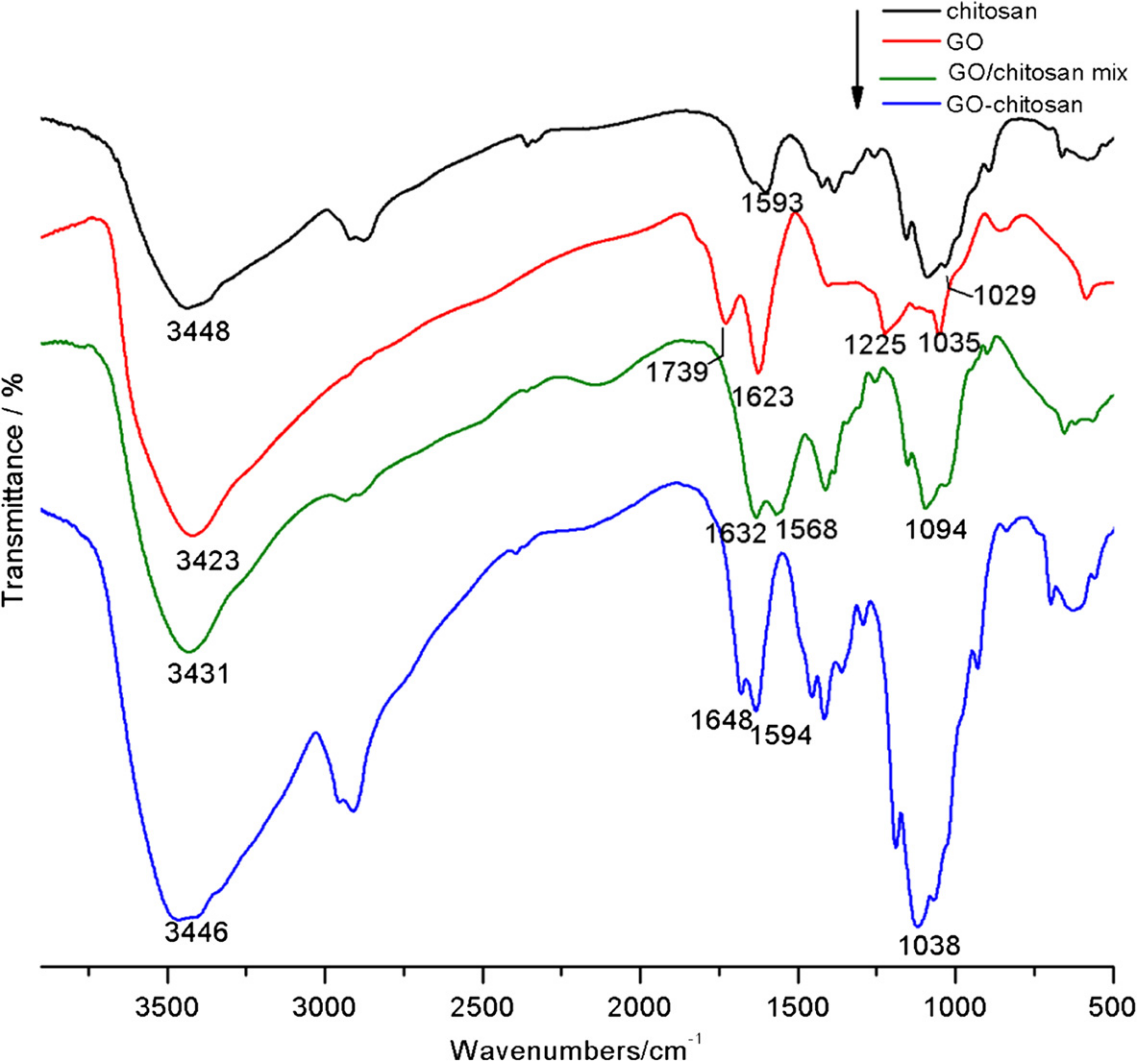
FTIR spectra of chitosan, GO, GO-chitosan and GO/chitosan mix.

Fourier transform infrared spectroscopy (FTIR) was used to verify the amide linkage between GO and chitosan. Spectra of the samples are compared in Figure 2. In the spectrum of GO, we observe dominant peaks are at 1035,1225,1623 and 1739 cm^-1^. The peak at 1035 cm^-1^ corresponds to a stretching vibration from C-O-Cbonds of epoxy or alkoxy. The peak at 1225 cm^-1^ is attributed to the C-OH bonds. While the peak centered at 1623 cm^-1^ is assigned to C=C bonds associated with skeletal vibrations of unoxidized graphite domains. The peak located at 1739 cm^-1^ is attributed to C=O in carboxylic acid and carbonyl moieties [25]. In the spectrum of chitosan, dominant peaks exist at 1029 cm^-1^ and 1593 cm^-1^. These peaks correspond to an absorbance of glucosidic bond, stretching vibration from C=O of-NHCO-and the N-H bending of NH_2_, respectively [26]. In the spectra of GO-chitosan, the dominant peaks at 1038 cm^-1^ and 1594 cm^-1^ correspond to the absorbance of glucosidic bond, stretching vibration from C=O of -NHCO- and the N-H bending of NH_2_, respectively, compared with pure chitosan and GO. In the GO-chitosan spectrum and GO/chitosan mix spectrum, the absence of the peak at 1739 cm^-1^, corresponding to C=O in carboxylic acid and carbonyl moieties in GO,is simply for the mass ratio of GO to chitosan is 1 to 9 in both GO-chitosan and GO/chitosan mix, thus the peak is too weak to be observed.

**Figure 3:**
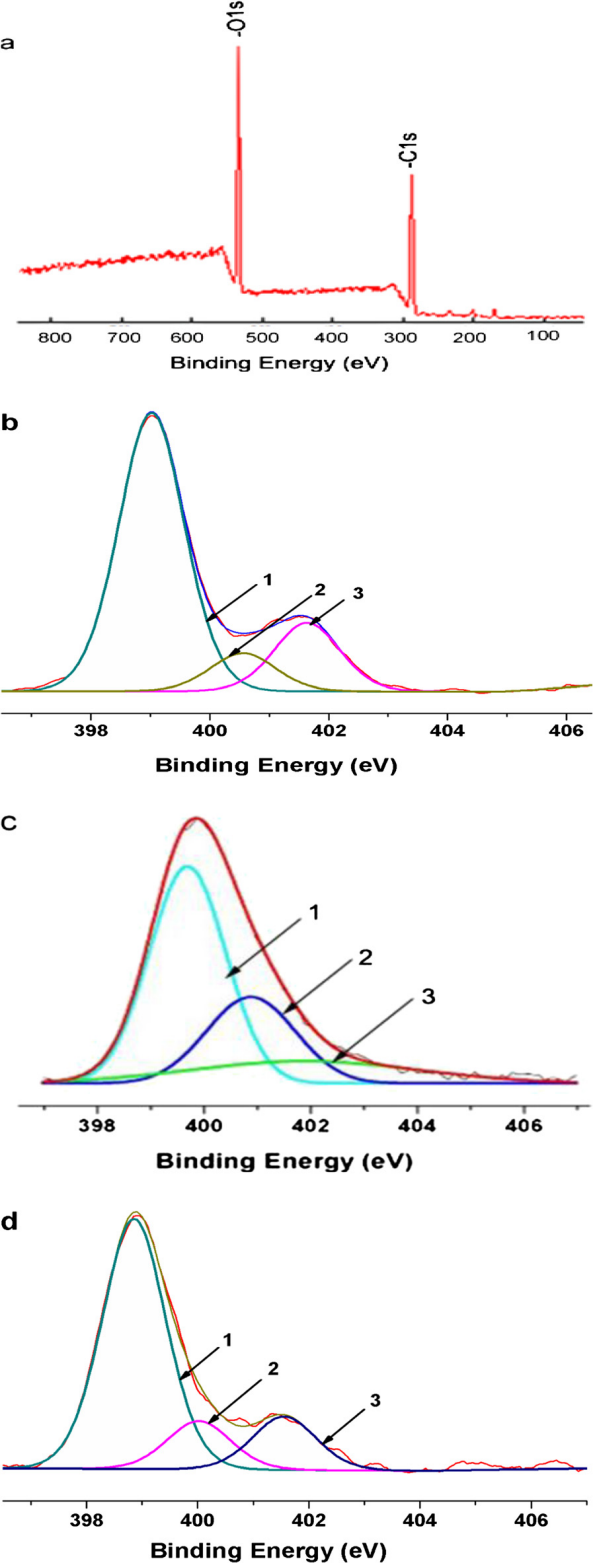
**XPS N1s core-level spectra of (a) GO, (b) chitosan, (c) GO-chitosan and (d) GO/chitosan mix.** The peaks 1, 2 and 3 correspond to C-NH_2_, C-NH-C=O, C-N^+^, respectively.

Following FTIR analysis, we used XPS to further explore the interactions between GO and chitosan. The survey of GO (Figure 3a) shows that no detectable amount of N1s, for the strongest XPS band of N-is usually found between 400 and 407 eV depending on the chemical environment [26]. Figure 3b shows the N1s XPS spectra of chitosan, with three different peaks centered at 399.0, 400.5 and 401.6 eV. These peaks correspond to C-NH_2_, C-NHC=O, and C-N^+^, respectively [27,28]. Compared with the pristine GO, the survey (Figure 3c) of GO-chitosan shows the presence of N1s originating from chitosan. The N1s spectrum of GO-chitosan (Figure 3c) can be convoluted into three peak components with binding energies at 399.4, 400.7, and 401.7 eV [28], attributed to the amine, amide, and the protonated amine species, respectively. Compared with chitosan, the relative increase in amide peak and decrease in protonated amine in GO-chitosan provides the supporting evidence for the formation of new covalent functionalization between the carboxylic acid on the surface of GO and N groups of chitosan. These data are consistent with FTIR. Additionally, we observe a marginal change in the relative heights of peaks 2 and 3, which indicates few new covalent bonds formed in the GO/chitosanmix (Figure 3d).

**Figure 4:**
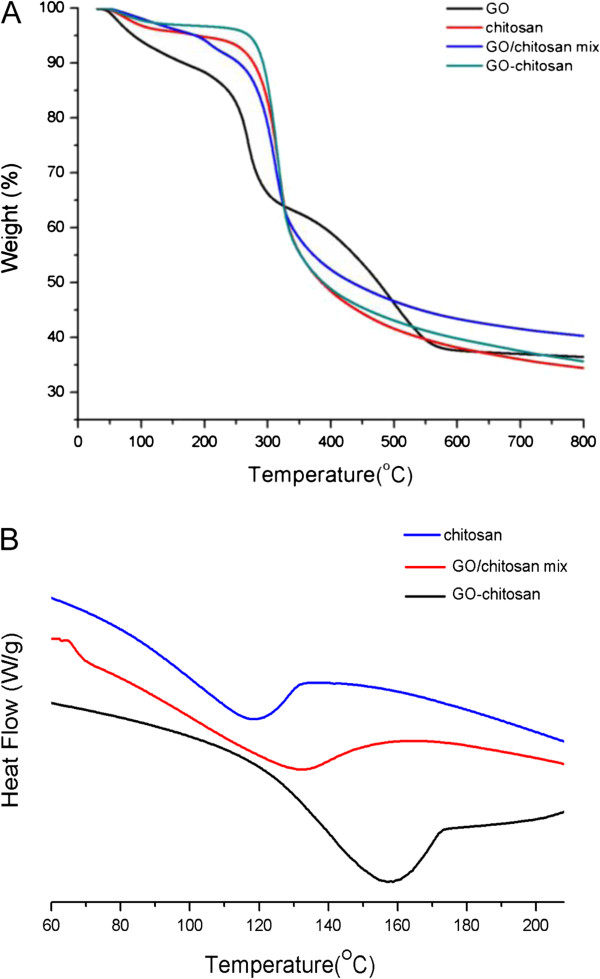
**Thermal properties of the composites. a** TGA curves ofGO, chitosan, GO/chitosan mix and GO-chitosan. **b** DSC curves to determine the Tg of chitosan, GO/chitosan mix and GO-chitosan.

Thermal properties of the composite films were evaluated with thermal gravimetric analysis (TGA) and differential scanning calorimetry (DSC). TGA reveals the composition and changes in thermal stability of the tested samples. As shown in Figure 4a, GO powder is thermally unstable and starts to lose functional groups below 100°C, and the major loss occurs at 250°C, presumably due to pyrolysis of the labile oxygen-containing functional groups, yielding CO, CO_2_ and water [29]. Because both GO-chitosan film and GO/chitosan film are mainly composed of chitosan (GO:chitosan = 1:9, m/m), their TGA curves are similar to that of the pristine chitosan film and do not have the obviously second major weight loss corresponding to decomposition of GO. Additionally, GO-chitosan is more thermally stable than GO. Apart from a slight mass loss below 150°C, which can be contributed to loss of water contained in the GO-chitosan film, no significant loss of mass is detected even at 300°C. The major loss of mass occurs at approximately 300°C, which is similar to that of chitosan. The chemical modification of thermally labile oxygen-containing functional groups of GO results in significantly increased thermal stability for the GO-chitosan.

The improvement in the thermal stability of the GO-chitosan composite can also be evidenced by the glass transition temperatures (Tg) in Figure 4b. The glass transition occurs at 118–119°C for pristine chitosan, whereas it shifts to 132°C for GO/chitosan mixture and 158°C for GO-chitosan composites. Both GO-chitosan and GO/chitosan mix have a rise in the Tg. Since Tg of polymers is affected by the mobility of polymer chains, the increasing of Tg and the thermal stability of the composites as compared to pristine chitosan could be inferred that evenly dispersion of GO might effectively hinder the motion of the chitosan chains by electrostatic attraction and hydrogen bonding, as demonstrated in previous reports [30]. Moreover, the chemical linkage between chitosan chains and GO sheets in GO-chitosan composite films might restrict the conformations of polymer molecules in a higher degree than the intermolecular forces, resulting in higher Tg of GO-chitosan composite films than that of GO/chitosan mixture.

**Figure 5:**
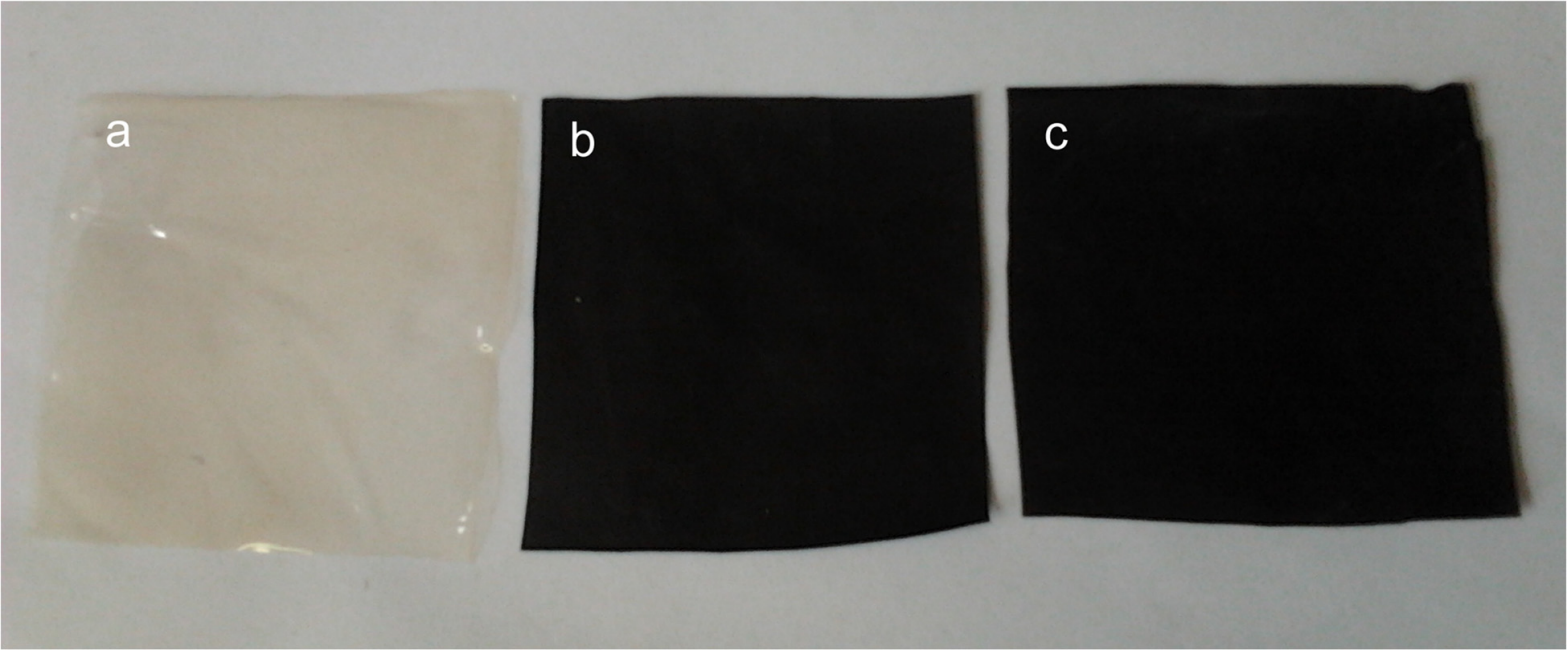
The photos of (a) chitosan film, (b) GO-chitosan film and (c) GO/chitosanmix film.

**Figure 6:**
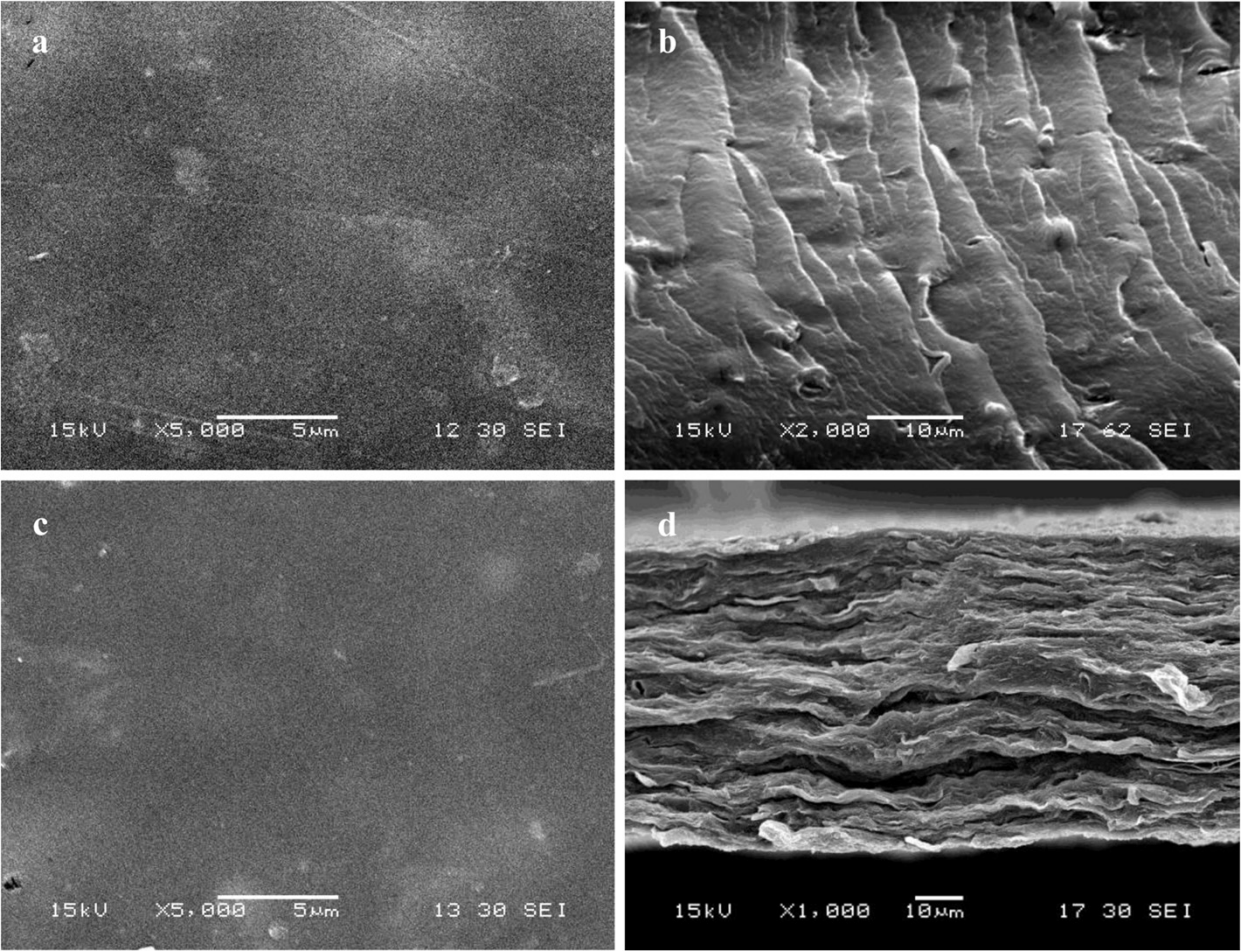
**SEM images of GO-chitosan and GO/chitosanmix films, respectively. a**. GO-chitosan film-surface image; **b**. GO-chitosan film fracture-surface image; **c**. GO/chitosan mix film-surface image; **d**. GO/chitosan mix film fracture-surface image.

Figure 5 shows the photographs of the prepared films. To determine the morphology of GO-chitosan film and GO/chitosan film, we used scanning electron microscopy (SEM). GO-chitosan film in Figure 6a is homogeneous and the fracture-surface image of the film in Figure 6b exhibits no stacking of GO. In contrast, the surface of GO/chitosan in Figure 6c film appears relatively coarse. Some protuberances can be observed in Figure 6c, since GO could aggregate during preparation of composite films. Similar results have been widely observed in the recent studies on polymer-immobilized graphene sheets [31,32]. GO-chitosan film and GO/chitosan film have a "sandwich" structure (Figure 6b and d). Figure 6b demonstrates that most of the GO nanosheets were fully exfoliated and clearly well dispersed in the chitosan matrix. Figure 6d shows that GO sheets are unidirectionally dispersed in the chitosan matrix and parallel to the composite film. Formation of amide linkages between GO and chitosan could facilitate dispersion of GO within the chitosan matrix and thus influence the mechanical properties of the resulting composites, which will be discussed in the following.

**Figure 7:**
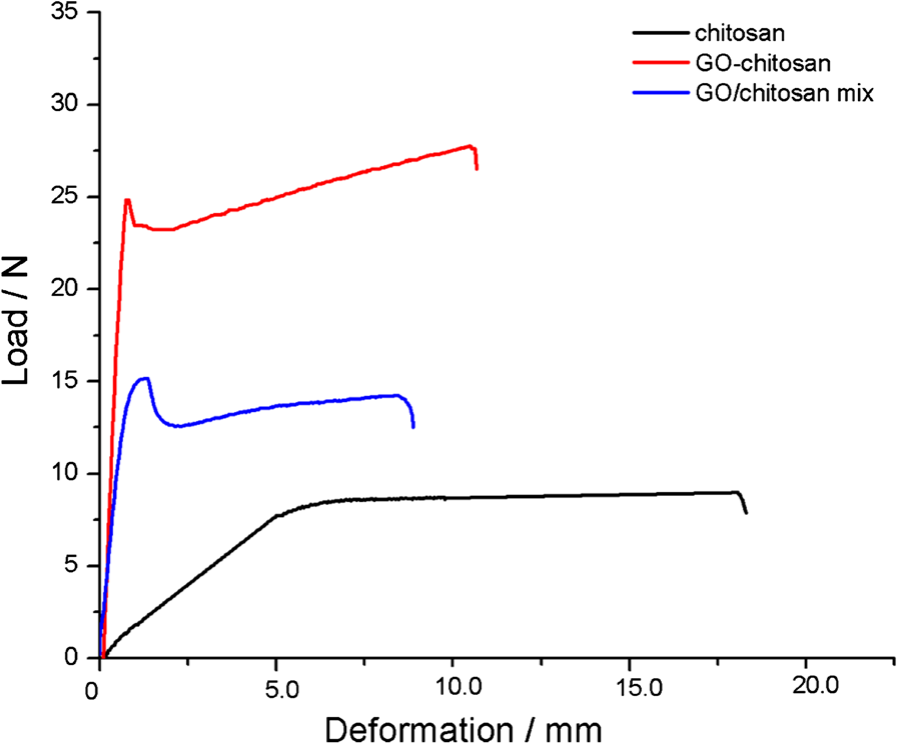
Tensile behaviors of the films.

The mechanical properties of the pristine chitosan, GO/chitosan and the GO-chitosan films were investigated by mechanical analyser (ZWICK ZO 20/TN2S, Germany) at 20°C. Mechanical properties of these films are summarized in Table 1, which are in good agreement with their thermal description. GO-chitosan is more thermally stable than GO/chitosan and GO-chitosan also performances better than GO/chitosan in mechanical properties. The strong chemical interaction between GO and chitosan and homogeneous dispersion of GO-chitosan result in a uniform stress distribution and be able to minimize the occurrence of stress concentration, leading to a significant increase in mechanical properties of the resulting nanocomposites. The uniform dispersion, together with the strong interfacial adhesion between GO and chitosan matrix, also enhances the mechanical properties of GO/chitosan mix film. Although the loading amount of GO in all GO-chitosan films was not very high, it significantly improves the mechanical properties. Figure 7 shows the relationship between the peak load and deformation of the films. Compared with the pristine chitosan film, the tensile strength of GO-chitosan film increases by 2.5 folds, from 32.7 to 82.0 MPa, the tensile strength of GO/chitosan mix film increases by 1.4folds, from 32.7 to 43.8 MPa. Besides, the addition of GO reduces the elongation at the break point. This could be attributed to the interaction between GO and the polymer matrix, which restricts the regular movement of chitosan chains [33]. Therefore, the Young’s moduli of GO-chitosan film and GO/chitosan mix film, as shown in Table 1, nearly increase by 4.6 folds and 2.1 folds than that of pristine chitosan film, respectively. Table 1 also shows that GO-chitosan film has a better mechanical performance than GO/chitosan mix film. Some researchers [33] assumed that a few GO restacked due to the van der Waals force and its surface area ratio reduced after certain level of GO loading. Beyond this critical loading limit, further increasing GO content could cause aggregation of GO with the polymer matrix and thus effect the mechanical improvement of chitosan films. So compared with the recent similar studies, we can infer that the GO content in GO/chitosan mix film may be beyond the critical loading and have less effective enhancement during the tensile testing.

**Table 1:** Mechanical properties of films

**Film sample**	**PeakLoad F**_**b**_**/N**	**Tensile strength σ**_**b**_**/MPa**	**Young's modulus E/MPa**	**Elongation at breakδ/%**
chitosan	9.1	32.7	447.7	71.3
GO/chitosan	15.2	43.8	1037.6	32.8
GO-chitosan	24.8	82.0	2090.5	42.5

Overall, compared with chitosan film, the increase in thermal and tensile behaviors of GO-chitosan film and GO/chitosan mix film could be ascribed to the loading of GO. On one hand, the reinforcing effect is partly because of the hydrogen bonding between chitosan and GO at the molecular level [34]. On the other hand, the polycationic nature of chitosan and the anionic nature of GO leads to electrostatic attraction between them also reducing the chain segmental mobility. Besides, GO-chitosan film achieves better thermal and tensile properties compared with GO/chitosan mix film. The amide linkage between GO and chitosan in GO-chitosan could contribute to the higher degree of restriction in mobility of the chitosan chains and thus lead to the stronger enhanced thermal and tensile properties.

Since chitosan has been widely studied in making biomedical materials, we explore here if the incorporation of GO could influence the biocompatibility of the chitosan with the mouse mesenchymalstem C3H10T1/2. For the quantity of cells adhered to the materials could not be observed by optical microscope due to the poor light-admitting quality of GO-chitosan films, the results were measured by tracing the nucleus of cells with DAPI. Morphology of cells was shown by double fluorescence staining. DAPI is a fluorescent dye that can penetrate the cell membrane and integrate tightly with DNA. Thus, it is used to fluorescently stain the nuclei of cells. Phalloidine is a kind of cyclic peptides yielded by Amanita phalloides. It can combine with filamentous-actin protein and often be used to stain the actin cytoskeleton of cells.

**Figure 8:**
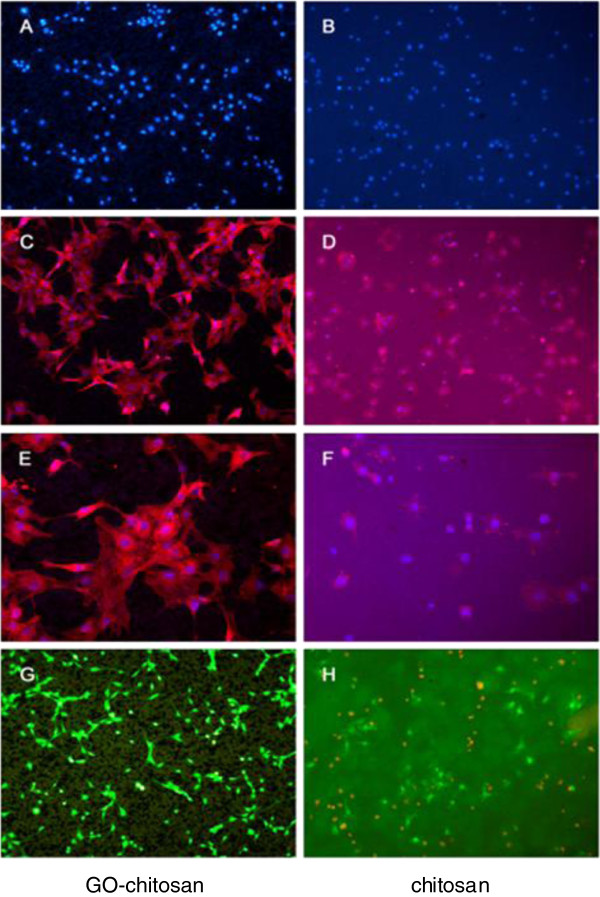
**Cell contrast fluorescence micrographs on GO-chitosan film and chitosan film.** Cell phase contrast micrographs onGO-chitosan film (**a**) and on chitosan film (**b**) at 24 h. (**c**) shows the cells adhering on the GO-chitosan film at 24 hunder 5Xobjective. (**d**) shows the cells adhering on the chitosan film at 24 hunder 5X objective. (**e**) and (**f**) are higher zoom of (**c**) and (**d**), respectively. In the above morphology of cells, the actin stains with phalloidinin red fluorescence, while nuclei stains with DAPI in blue fluorescence. A cell viability stain on GO-chitosan film (**g**) and on the chitosan film (**h**), respectively. The assays kit produces a green fluorescence in live cells while a bright red fluorescence in dead cells.

Figure 8 shows the morphology of cells with blue DAPI stain for nucleiand red phalloidine stain for actin. After 24 h of culture, cell proliferation was observed on both the chitosan film and GO-chitosan film, and the morphology of these cells showed no obvious variances between them (Figure 8a and b). Figure 8c shows the cells adhering onto the GO-chitosan film under 5× objective. Figure 8d shows the cells adhering on the chitosan film under 5× objective. Figure 8e and f are higher zoom of Figure 8c and d, respectively. Based on cell fluorescence, GO-chitosan film is as good as chitosan film for cells adhesion. The proliferation of cells on two samples indicated that both of them had very little cytotoxicity and could have a potential for stimulating interaction between cells and the films, which could serve as an ideal platforms for cell adhesion and proliferation.

A cell viability stain was also used to test the biocompatibility of the films. The assays kit produced an intense uniform green fluorescence in live cells, while a bright red fluorescence in dead cells was observed. After 24 h, cells on the surface of GO-chitosan film showed better viability as observed by green fluorescent staining (Figure 8g). There were large amount of dead cells with red fluorescence on the chitosan film (Figure 8h). The results indicate that the GO-chitosan film is suitable for cell proliferation.

Magrez et al. [35] explored the influence of surface functionalities on the toxicity of carbon-based nanomaterials (CBNs) and found out that the toxicity increased as the oxygen containing groups were introduced onto the surface of CBNs. Thus, it is intriguing to know the toxicity of GO-chitosan film with the C=O, OH, and COOH groups on the surfaces. However, in comparison with CBNs [36-39], these hydrophilic groups increase the solubility and dispersion of GO-chitosan, and may have limitedly negative effects on cells after chitosan is decomposed in the body. Hence, GO-chitosan film has an acceptable biocompatibility in a limited GO content range, providing an alternative in functional biomaterial.

## Conclusions

The present study describes a useful and simple method to chemically attach biocompatible chitosan onto graphene oxide through the formation of amide linkages between GO’s carboxylic acid groups and chitosan's amine groups. GO-chitosan composite films were also characterized by element analysis, Fourier transform infrared spectroscopy, X-ray photo electron spectroscopy, differential scanning calorimetry, and thermo gravimetric analysis. Both the tensile strength and Young’s modulus of GO-chitosan film increase dramatically. GO-chitosan is more thermally stable than chitosan. Meanwhile, GO-chitosan film has good biocompatibility, biodegradability, and good solubility in aqueous medium, similarly to chitosan. The results of both cell proliferation and cell viability experiments indicate that GO-chitosan film is suitable for cell proliferation. Taken together, we envision that the GO-chitosan film will open avenues for next-generation graphene applications in the realm of functional biomaterial.

## Methods

### Materials

Chitosan (reagent grade) was supplied by Shanghai Weikang Biological Co. (China) with a degree of deacetylation of 93% and average molecular weight of 400,000 g/mol. Graphite powder was purchased from Shanghai Yi Fan Co., China. Sulphuric acid (H_2_SO_4_, 98.0%), potassium permanganate (KMnO_4_, 99.9%), hydrogen peroxide (H_2_O_2_, 30.0%), Phosphate standard concentrate (H_3_PO_4_, 85.0%), Hydrochloric acid (HCl, 37.0%) and acetic acid (99.9%) were purchased from Shanghai Lingfeng Chemical Reagent Co.,China. *N,N*-dimethylformamide (DMF, 99.8% SuperDry, with molecular sieves)was purchased from J&K Chemical (Beijing) Ltd.,China. *N,N'*-dicyclohexylcarbodiimide (DCC, 99.0%), 1,2-dichlorobenzene (ODCB, 99.0%) and 4-dimethylaminopyridine (DMAP,99.0%) were purchased from Aladdin Reagent (Shanghai) Co., Ltd, China. The reagents were used without further purification.C3H10T1/2 cells were provided by the Chinese Academy of Sciences (Shanghai, China). Dulbecco's minimum essential medium (DMEM) and Penicillin and streptomycin were purchased from Thermo (USA). TritonX-100 solution, phalloidine and 4',6-diamidino-2-phenylindole (DAPI)were purchased from Sigma (USA). LIVE/DEAD viability/cytotoxicity Assay Kit and Fetal Bovine Serum were purchased from Life Technologies (USA).

### Synthesis of graphene oxide-chitosan (GO-chitosan)

Graphene oxide (GO) was prepared according to Hummers’method [40]. Briefly, 20 mL H_3_PO_4_ (85.0%) and 180 mL of concentrated H_2_SO_4_ were poured into the three-neck flask charged with 1 g graphite powder under stirring. 6 g KMnO_4_ was gradually added into the reaction mixture that was cooled by an ice bath. The cooling bath was removed and the reaction mixture was kept at 45°C for 48 h to ensure complete oxidation of graphite. The reaction mixture was quenched by 1 L of ice containing 10 mL of 30% H_2_O_2_. The color of the mixture changed from dark brown to bright yellow, indicating formation of GO. The solid was obtained by centrifugal separation and washed 3 times with 10% HCl aqueous solution followed by deionized water until pH of 5–6 was achieved. Finally, the resulting GO was washed with acetone and then dried at 60°C under vacuum for 30 h.

**Scheme 1:**
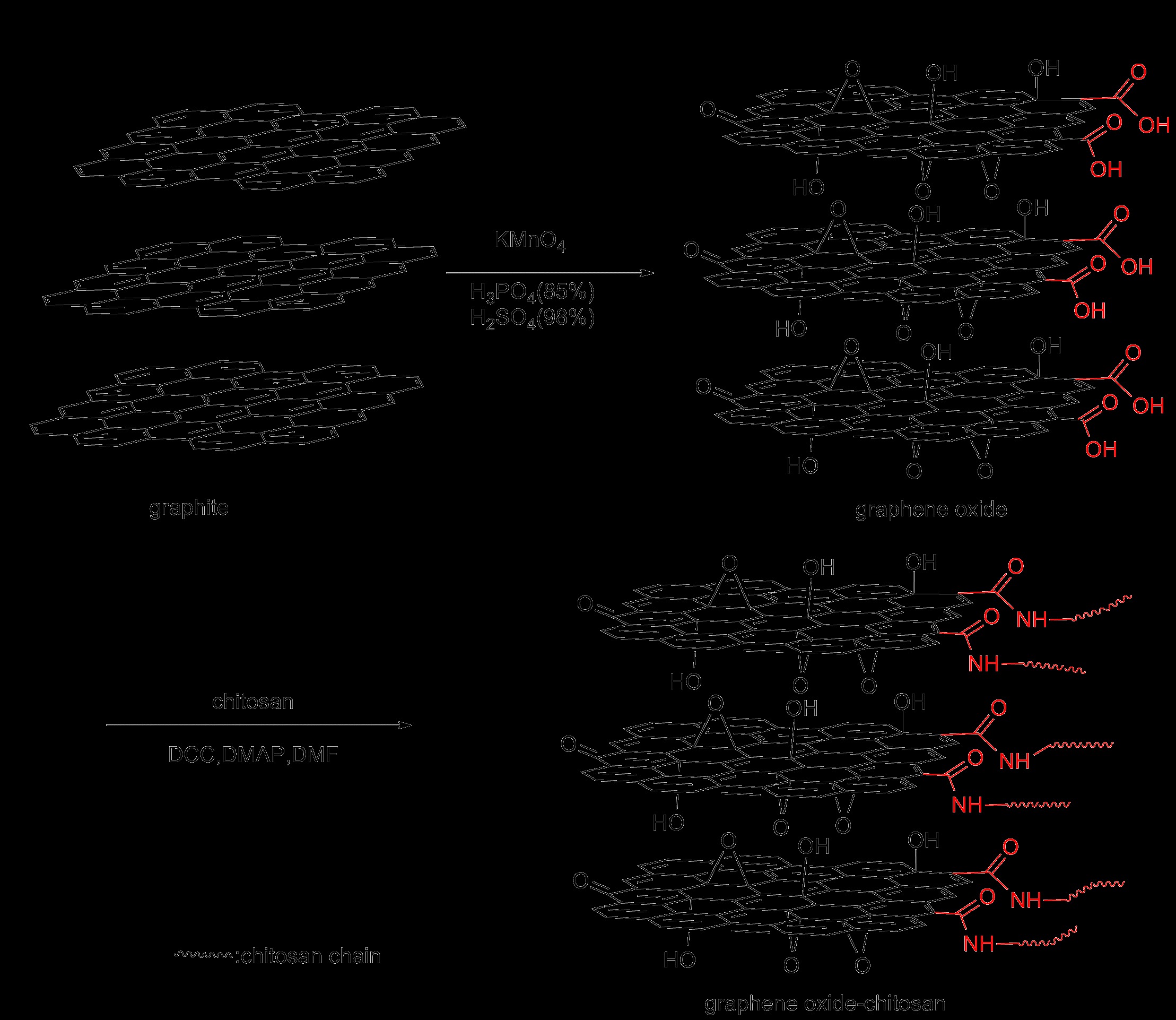
Schematic diagram to produce GO-chitosan.

Graphene oxide-chitosan composites were prepared as follows: 50 mg GO and 1gchitosan were loaded into a 100 mL round-bottom flask charged with 50 mL DMF. The mixture was sonicated for 1h, 0.45 g DCC and 0.3 g DMAP were then added to the above suspension and incubated for 48 h at room temperature. The resulting solid was isolated by centrifugation and washed with ODCB (3×50 mL) to remove unreacted chitosan. The mixture was subsequently washed thoroughly with water (50 mL), methanol (50 mL) and acetone (50 mL), sequentially. Finally, it was dried at 60°C for 24 h under vacuum. The fabrication process is outlined in Scheme 1.

### Fabrication of chitosan film

500 mg chitosan powders were dissolved in 25 mL 2% (v/v) acetic acid aqueous solution upon stirring at 200rpm for 3 h. After that, the chitosan suspension was poured into a plastic dish that was placed in a fume hood at room temperature to evaporate water and acetic acid. The as-prepared composite film was dried under vacuum at 40°C until their weight was equilibrated.

### Fabrication of graphene oxide-chitosan (GO-chitosan) film

500 mg graphene oxide-chitosan powders were dissolved in 25 mL 2% (v/v) acetic acid aqueous solution by ultrasonication for 30 min. The homogeneous solution was then cast onto a teflon plate, left to dry at room temperature and subsequently peeled off as a free-standing film. The film was dried under vacuum at 40°C until its weight was equilibrated.

### Fabrication of graphene oxide/chitosanmix (GO/chitosanmix) film

0.25wt% GO solution was prepared by dissolving 100 mg GO in 40 g water and treated with ultrasonication for 30 min. Specific amounts of chitosan powders were then added into the GO solution, followed by ultrasound for 1h. The solution was then stirred at 200 rpm for 3 h. After stirring, the GO/chitosan suspension was poured into a flat dish, placed in fume hood at room temperature, allowing water to evaporate for film formation. Then, the film was stored under vacuum at 40°C until its weight equilibrated.

Overall, the volume of each casting solution was controlled to be 18.2 mL. The size of all the flat dishes for film formation was 87 mm × 87 mm in order that all the films were in the same size. The films were taken at random and each film was cut into 6 small pieces of 25 mm × 25 mm in size. The average mass of each small piece of chitosan film, GO-chitosan film and GO/chitosan mix film was 0.128 g, 0.137 g and 0.132 g, respectively. The standard deviation of each kind of film was 4.86×10^-3^,4.14×10^-3^ and 4.02×10^-3^, correspondingly, which proved that the solution-casting method was feasible and the composite films were uniform.

### Characterizations

Element analysis was carried out on Elementar Vario EL III (Germany). Fourier-transform infrared spectra (FTIR) were recorded on a Nicolet FTIR Infrared Microscopy. X-ray photoelectron spectroscopy (XPS) measurements were performed using a PHI Quantera XPS with a monochromated Al K*α* radiation (hν=1486.6eV). Thermal properties of the films were studied by thermal gravimetric analysis (TGA) and differential scanning calorimetry (DSC). TGA was performed under a nitrogen flow (60 mL/min), and the weight was recorded as a function of temperature. DSC was measured by a TA 2910 (USA) at heating rate 20°C/min under N_2_ at a flow rate of 80 mL/min. Scanning electron microscopy (SEM)images were taken on a FEI Quanta 400 ESEM FEG. The mechanical characterization was performed on a mechanical analyser (ZWICK ZO 20/TN2S, Germany). Each film was cut into five dumbbell strips with a size of 75 mm × 4 mm. The lower grip was fixed and the upper grip rose at an extension rate of 10mm/min with a preload of 1.0 N. All the failures occurred at the middle region of the testing strips.

### Biocompatibility of graphene oxide-chitosan (GO-chitosan) film

#### Cell culturing

Mouse mesenchymal Stem C3H10T1/2 cells were cultured in Dulbecco's minimum essential medium (DMEM) supplemented with 10% Fetal Bovine Serum and a combination of 100 ug/mL penicillin and 100 ug/mL streptomycin. All incubations were at 37°C with a concentration of 5% CO_2_.

#### Proliferation and viability of C3H10T1/2 cells

Fluorescent staining method was used in analyzing the proliferation of cells on the surface of GO-chitosan films and chitosan films. Firstly, GO-chitosan films and chitosan films were sterilized with cyclohexane, followed by immersion in DMEM seeded with a concentration of 5×10^6^ cells/mL at 37°C. Briefly, after being cultured for 24 h, the samples were gently washed with phosphatebuffered saline (PBS) and maintained in 4% paraformaldehyde for 15 min. This step was followed by being immersed in 0.1%TritonX-100 solution for 15 min, and washed with PBS again. The samples were stained in a new 24-well plate with 500 μLphalloidine for 1 h, and then stained with 4',6-diamidino-2-phenylindole (DAPI) for 15 min.C3H10T1/2 cells seeding on films and staining was done according to the manufacturer’s instruction. Stained cells were then ready for observation through fluorescence microscope (Leica DM4000B, Germany).

A LIVE/DEAD viability/cytotoxicity Assay Kit was also used to investigate the viability of cells. Samples were prepared as described above. After C3H10T1/2 cells were cultured on the surface of GO-chitosan films and chitosan films for 24 h, the samples were gently washed three times with PBS, and then stained in another new 24-well plate with 500 μL stain solution for 45 min. The viability of cells was observed using fluorescence microscope.

## Abbreviations

GO: Graphene oxide; DMF: *N,N*-dimethylformamide; DCC: *N,N'*-dicyclohexylcarbodiimide; DMAP: 4-dimethylaminopyridine; ODCB: 1,2-dichlorobenzene; DMEM: Dulbecco's minimum essential medium; PBS: Phosphatebuffered saline; DAPI: 4',6-diamidino-2-phenylindole; FTIR: Fourier-transform infrared spectra; XPS: X-ray photoelectron spectroscopy; TGA: Thermal gravimetric analysis; DSC: Differential scanning calorimetry; Tg: Glass transition temperature; SEM: Scanning electron microscopy.

## Competing interests

The authors declare that they have no competing interests.

## Authors’ contributions

PPZ carried out the experiment and prepared the manuscript for submission. HFF participated in the study of biocompatibility of graphene oxide-chitosan film. ZZX participated in solving the problems in synthesizing. LFZ conducted the spectroscopic analysis. YLZ participated in the results discussion. WX interpreted the spectral data and revised the manuscript. WQZ proposed the research idea and coordinated final formulation. All authors read and approved the final manuscript.

## Authors’ information

Shanghai Key Laboratory of Functional Materials Chemistry, School of Chemistry and Molecular Engineering, East China University of Science and Technology, 130 Meilong Road, Shanghai, China 200237.
